# First report on the serum chemistry and haematology of free-ranging dusky (*Carcharhinus obscurus*) and sandbar (*Carcharhinus plumbeus*) sharks in the eastern Mediterranean Sea

**DOI:** 10.1093/conphys/coad037

**Published:** 2023-05-30

**Authors:** Tal Starostinetsky-Malonek, Aviad Scheinin, Itamar Aroch, Nadav Davidovich, Eyal Bigal, Leigh Livne, Rachel Ann Hauser-Davis, Natascha Wosnick, Dan Tchernov, Danny Morick

**Affiliations:** Morris Kahn Marine Research Station, Leon H Charney School of Marine Sciences, University of Haifa, Sdot Yam 3780400, Israel; Morris Kahn Marine Research Station, Leon H Charney School of Marine Sciences, University of Haifa, Sdot Yam 3780400, Israel; School of Veterinary Medicine, Hebrew University of Jerusalem, PO Box 12, Rehovot 7610001, Israel; Morris Kahn Marine Research Station, Leon H Charney School of Marine Sciences, University of Haifa, Sdot Yam 3780400, Israel; Israeli Veterinary Services, Bet Dagan 5025001, Israel; Morris Kahn Marine Research Station, Leon H Charney School of Marine Sciences, University of Haifa, Sdot Yam 3780400, Israel; Morris Kahn Marine Research Station, Leon H Charney School of Marine Sciences, University of Haifa, Sdot Yam 3780400, Israel; Laboratório de Avaliação e Promoção da Saúde Ambiental, Instituto Oswaldo Cruz, Fundação Oswaldo Cruz, Av. Brasil, 4.365, Manguinhos, Rio de Janeiro 21040-360, Brazil; Programa de Pós-Graduação em Zoologia, Universidade Federal do Paraná, Caixa Postal 19031, Curitiba, Paraná 81531-980, Brazil; Morris Kahn Marine Research Station, Leon H Charney School of Marine Sciences, University of Haifa, Sdot Yam 3780400, Israel; Morris Kahn Marine Research Station, Leon H Charney School of Marine Sciences, University of Haifa, Sdot Yam 3780400, Israel; Hong Kong Branch of Southern Marine Science and Engineering Guangdong Laboratory (Guangzhou), Kowloon, Hong Kong 999077, China

**Keywords:** shark, serum chemistry, Mediterranean Sea, haematology, Carcharhinus plumbeus, Carcharhinus obscurus

## Abstract

Shark assessments in the Mediterranean Sea are still scarce, and serum chemistry and haematological data have yet to be reported for wild dusky (*Carcharhinus obscurus*) or sandbar (*Carcharhinus plumbeus*) shark populations in the Mediterranean Sea. Herein, blood samples were obtained from adult dusky (*n* = 23) and sandbar (*n* = 14) sharks from an aggregation site near the Hadera power and desalination plants in Israel in the winters of 2016–20. Several serum chemistry analytes were characterized with relation to stress, body size and environmental conditions. Glucose concentrations were higher, while total cholesterol concentrations were lower in dusky sharks than in sandbar sharks, potentially due to distinct metabolic pathways utilized during the capture-related activity by both species. However, differences in sex and size are noted and should be considered. The blood cell morphology of both species was consistent with previous findings for sandbar sharks. Atypical monocytes were noted in one dusky shark. Preliminary and exploratory reference intervals for female dusky sharks were calculated for glucose, triglycerides, total cholesterol, total protein and creatine kinase. These data must be viewed with caution due to the potential influence of capture-related stress on analyte concentrations and activities and the fact that only females were employed in the calculations. Moreover, the sampling site is adjacent to coastal power and desalination plants, which may significantly affect shark physiology. Although limited, this novel database on dusky and sandbar shark serum chemistry and haematology aspects is essential as a first attempt to obtain data on these species in the eastern Mediterranean Sea and for future conservation and long-term biomonitoring efforts.

## Teaser

A novel database on the serum chemistry and hematology of adult free-ranging female dusky (Carcharhinus obscurus) and male sandbar (C. plumbeus) sharks from a coastal power and desalination plants aggregation site in the eastern Mediterranean Sea. Analyte relations to environmental and temporal parameters, body size, species, and stress were characterized.

## Introduction

The Mediterranean Sea is the largest and deepest enclosed sea on our planet, comprising a marine biodiversity hotspot with a high degree of endemism and harbouring an array of emblematic threatened species (Coll *et al.,* 2010). It is also home to several ecologically important, large keystone elasmobranch species, mostly apex predators that play critical roles in the structure and functioning of marine ecosystems (Stevens *et al.,* 2000; Coll *et al.,* 2013). However, despite their importance, information on many Mediterranean Sea keystone species, such as sharks, is still extremely limited. Biological and physiological studies concerning these animals in this area are, thus, paramount to improve management and conservation efforts and enable ecosystem health monitoring.

Elasmobranchs (sharks and rays) are generally characterized by relative longevity, late sexual maturity and a low number of offspring. These traits make this group highly susceptible to anthropogenic impacts, including, but not limited to, fisheries activities, habitat degradation and chemical pollution. The recovery of their populations is exceptionally prolonged and arduous (Cortés, 2004; Dulvy *et al.,* 2021). As a result, recent estimates indicate that 32.6% of the world's chondrichthyans are threatened, at a considerably higher extinction risk than most other vertebrates, including both large oceanic and coastal species (Dulvy *et al.,* 2021; Pacoureau *et al.,* 2021), with population declines especially prevalent in the Mediterranean Sea (Dulvy *et al.,* 2014, 2021). In this regard, the Mediterranean and Black Seas, home to about 43 shark species (International Union for Conservation of Nature, IUCN, 2021), are categorized as the world's most hazardous areas for chondrichthyans (Cavanagh and Gibson, 2007; Abdul Malak *et al.,* 2011; Dulvy *et al.,* 2014). In fact, estimates indicate that 64.5% of Mediterranean Sea chondrichthyan species and almost 70% of Mediterranean Sea shark species are threatened, and only 13.1% and 7% of them, respectively, are considered safe (*i.e.* least concern) (IUCN, 2021). Furthermore, the Mediterranean populations of certain large predatory sharks have dwindled by as much as 96–99.99% over the past two centuries.

Historically, large coastal requiem sharks, such as the dusky (*Carcharhinus obscurus*) and the sandbar (*Carcharhinus plumbeus*) sharks, have been regularly targeted or caught as bycatch by fisheries in the Mediterranean Sea in the early 20th century. Because of this, sharp declines have been noted in most of the Mediterranean Sea for these sharks in the last 35–40 years (Ferretti *et al.,* 2008). Israel, however, is an exception, as requiem sharks are still frequently observed and caught by fishers in both shallow waters and offshore. This occurs even though Israeli law protects all shark species, and their fishing is illegal, as insufficient enforcement still enables occasional illegal shark fishing (Edelist and Rilov, 2014).

In this regard, several dozens of sharks, mainly comprising adult female dusky and adult male sandbar sharks (Zemah Shamir *et al.,* 2019), aggregate in the water outflows of power plants along the Israeli Mediterranean coastline in Hadera, Ashdod and Ashkelon every winter, seemingly linked to the increased water temperature around the power plants. This phenomenon has been observed for the past three decades and increased between 1993 and 2013 relative to the preceding 20-year period (Barash *et al.,* 2018). However, the proximity to the aforementioned power plants makes these sharks more susceptible to fishing exploitation and coastal pollution. This is significant, as both dusky and sandbar sharks are categorized globally by the IUCN as ‘endangered’, with the former classified as ‘data deficient’ in the Mediterranean Red List, where population trends are still unknown (IUCN, 2021), and the latter as ‘endangered’ in the Mediterranean Red List, presenting decreasing population trends (IUCN, 2021). Nevertheless, to date, little to no studies of sharks in the Israeli Mediterranean Sea are available, and quantitative data are scarce.

Serum chemistry assessments are valuable for health monitoring efforts in wild species, although serum chemistry may vary geographically with ecological habitats and between genetically distinct populations (Whiting *et al.,* 2007). Several blood variables, such as glucose, cholesterol and phosphorus, have been associated with elasmobranch capture stress, and their use may, for example, inform management strategies aimed at reducing the impacts of longline capture on sharks (Wosnick *et al.,* 2017; Bouyoucos *et al.,* 2018). Energy parameters (*e.g.* glucose) can inform on nutritional conditions and have been reported to exhibit seasonal variations in some species, thus comprising a valuable tool for conservation physiology (Gallagher *et al.,* 2017). Furthermore, salts directly influence blood pH through exercise intensity, acting against physiological impairment, and have been reported as higher in warmer conditions (Bouyoucos *et al.,* 2018). In this regard, plasma and serum chemistry analytes have been reported previously for both dusky (Cliff and Thurman, 1984; Marshall *et al.,* 2012) and sandbar (Spargo, 2001; Marshall *et al.,* 2012; Jerome *et al.,* 2017) populations in the western Pacific Ocean, eastern North Pacific Ocean, and western North Atlantic, while haematological data have been reported only for sandbar sharks (Arnold, 1997; Haines and Arnold, 2014). Reference intervals (RIs), defined as the 95% confidence interval (CI) of a healthy reference population, with 90% CIs of the limits, provide clinical baselines for clinical assessments, health trends in wild species and imperilled health states caused by anthropogenic or environmental factors (Yang *et al.,* 2019). Defined RIs in sharks, however, are still scarce, mainly due to low accessibility and handling difficulties (Otway, 2015). While the minimal desirable reference population size for establishing RIs is 120, preliminary RIs have been reported as possible to calculate using reference populations as small as 20 to 40 animals, using parametric or robust methods. However, the width of the 90% CIs of their limits is likely to exceed the recommended 20% of the RI width (Friedrichs *et al.,* 2012). As serum chemistry data and RIs are unavailable for any Mediterranean Sea shark population, the present study aimed to report novel haematological and serum chemistry data of free-ranging dusky and sandbar sharks in the Mediterranean Sea, which may be employed in future monitoring assessments.

## Materials and methods

### Shark sampling

The Morris Kahn Marine Research Station crew has been performing catch-and-release tagging of sharks aggregating near the Hadera power and desalination plants' outlet (32°27′53.8″N, 34°52′55.1″E) in winter (November–April) since 2016. Data on species, sex, somatic measurements [fork length (FL), considered a more precise measurement than total length (TL) (Dudley *et al.,* 2005)] and fishing efforts (date; capture, handling and release times; capture method; and geographic location) were obtained in each sampling. Temperature and salinity were measured at the bottom at drumline deployment points using a probe (YSI Pro1030; Yellow Spring Instruments Incorporated, Yellow Springs, OH). Sharks were captured using handlines comprising a baited hook attached to a large buoy or autonomous drumlines comprising a baited hook attached to a buoy and a weight. Upon capture, sharks were pulled to the boat side and restrained, and biometric data and caudal venipuncture blood samples (minimum, 4 mL; 10-mL syringe; 16-gauge, 1.75″ needle) were obtained (Walsh and Luer, 2004). Whole blood was placed in lithium–heparin and serum tubes with gel separators (Greiner Bio-One, Kremsmünster, Austria). Samples were kept upright in a cooling box for no more than 4 hours pending arrival to the research station.

### Blood analyses

Blood samples were centrifuged at 1750*g* for 3 minutes to obtain sera, which, alongside heparinized whole blood samples, were shipped chilled to a diagnostic laboratory at the Hebrew University Veterinary Teaching Hospital, Rishon LeZion, Israel. Serum chemistry analyses [urea, total protein, glucose, total cholesterol, triglycerides, calcium, phosphorus, chloride, sodium, potassium, total bilirubin, aspartate aminotransferase (AST), γ-glutamyl transpeptidase (GGT), amylase and creatine kinase (CK)] were performed using a wet chemistry autoanalyser (at 37°C, Cobas 6000; Roche, Mannheim, Germany).

Blood smears were prepared, air-dried and stained using a Hematek stainer (Siemens Medical Solutions, Solna, Sweden) using a modified Wright's staining solution. Additional blood smear sets were prepared from heparinized whole blood, air-dried and manually stained with a quick Romanowsky staining solution (JorVet Dip Quick Stain; Jorgensen Laboratories, Loveland, CO). The stained blood smears were used for microscopic manual differential leukocyte counts and blood cell morphology assessments. Packed cell volumes (PCVs) were determined by centrifuging heparinized whole blood in heparinized capillaries at 2570*g* for 3 minutes and 20 seconds and read manually using a reader card.

### Statistical analyses

In hemolysed sera, results of analytes expected to be affected and lead to false results (*e.g.* calcium, phosphorus, chloride, sodium and potassium) were excluded from the statistical analyses. When the measured GGT activity was below the minimum limit of detection (<3.0 U/L), its activity was set at 2.9 U/L for the purpose of statistical analyses. Suggested RIs were calculated using Reference Value Advisor 2.1 (Geffré *et al.,* 2011) according to the guidelines of the American Society for Veterinary Clinical Pathology (ASVCP) (Friedrichs *et al.,* 2012). The programme uses the standard or robust method according to Horn *et al.* (1998) and Box–Cox transformation, and provides 90% CI for the reference limits.

Statistical analyses were performed using R Version 4.0 software, stats R package (R Core Team, 2020). A *P* value ≤0.05 was considered significant for all analyses.

Data were tested for normality by the Shapiro–Wilk test and equality of variance by the *F* test. The two shark species were compared using the Student's *t*-test or Wilcoxon test for normally and non-normally distributed analytes, respectively. Other comparisons between two groups were performed using the Wilcoxon test. Comparisons of more than two groups were performed using the Kruskal–Wallis test, with *post hoc* comparison *P* values adjusted using the Benjamini–Hochberg method using the ‘dunn.test’ R package. Associations between variables were examined using the Spearman's rank correlation test. Finally, comparison of the present results with values from the literature was performed using one sample *t*-test for normally distributed data or one sample Wilcoxon test for non-normally distributed data.

For data analysis purposes, the sampling season was divided into two parts—beginning (November–January) and ending (February–April), and the time of day was divided into three parts—morning (06:30–10:30 h), noon (10:30–14:30 h) and afternoon (14:30–18:00 h).

## Results

### Shark samplings

A total of 23 dusky sharks (females, 22; males, 1) and 14 sandbar sharks (all males), all adults (dusky shark females, >257-cm TL; sandbar shark males, >130-cm TL; Serena, 2005), were sampled. Dusky sharks were larger (median FL, 245 cm; FL range, 215–343) than sandbar sharks (median FL, 150 cm; FL range, 139–160 cm) and spent longer times on the hook from capture to release (mean, 47 min; SD, 12, vs. mean, 33; SD 5, respectively). Detailed shark, fishing efforts and environmental data, along with laboratory results, are provided in Supplementary Table S1.

### Serum chemistry results

#### Serum chemistry interanalyte associations

**Figure 1:**
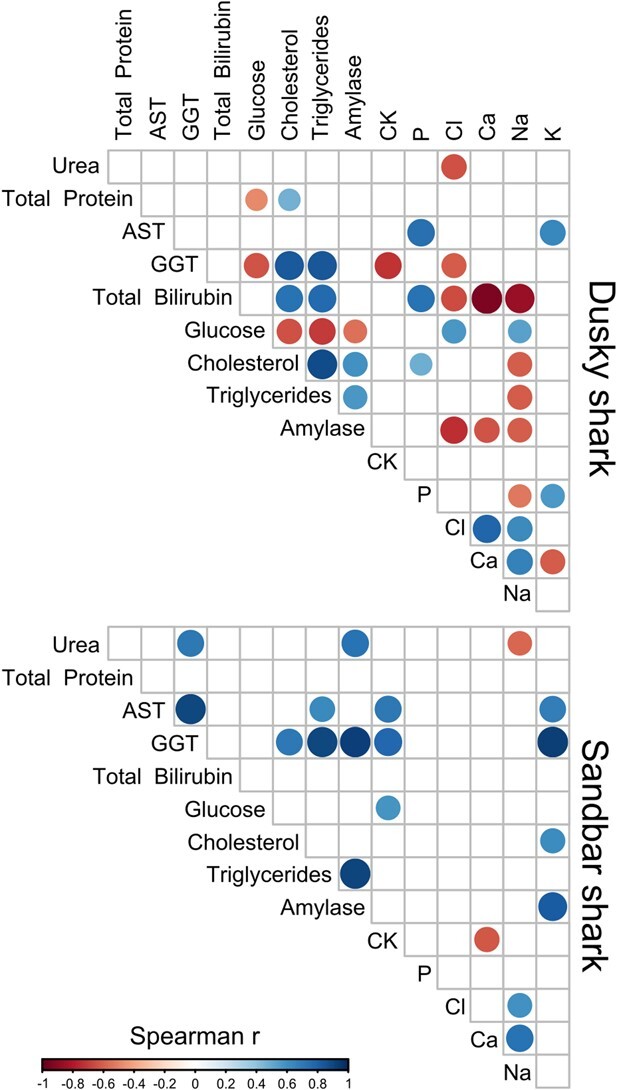
Statistically significant (*P* ≤ 0.05) Spearman's rank correlations between serum chemistry analytes in wild dusky (*C. obscurus*) and sandbar (*C. plumbeus*) sharks from the eastern Mediterranean Sea. Spearman's *ρ* is represented by circle size and colour with a colour-coded legend.

Correlations between the 15 determined serum chemistry analytes in both shark species are presented in Figure 1. Significant positive correlations were found in both shark species between triglycerides concentration and amylase activity (dusky, *ρ* = 0.57, *P* = 0.026; sandbar, *ρ* = 0.917, *P* = 0.001), triglycerides concentration and GGT activity (dusky, *ρ* = 0.84, *P* < 0.001; sandbar, *ρ* = 0.905, *P* = 0.001), GGT activity and total cholesterol concentration (dusky, *ρ* = 0.833, *P* < 0.001; sandbar, *ρ* = 0.705, *P* = 0.034), AST activity and potassium concentration (dusky, *ρ* = 0.648, *P* = 0.009; sandbar, *ρ* = 0.689, *P* = 0.028), sodium and chloride concentrations (dusky, *ρ* = 0.626, *P* = 0.005; sandbar, *ρ* = 0.592, *P* = 0.043) and sodium and calcium concentrations (dusky, *ρ* = 0.666, *P* = 0.004; sandbar: *ρ* = 0.736, *P* = 0.01). CK and GGT activities were negatively correlated in the dusky shark (*ρ* = −0.711, *P* = 0.004) but positively so in the sandbar shark (*ρ* = 0.774, *P* = 0.024). No other significant correlations were detected.

#### Serum chemistry analyte descriptive statistics and preliminary RIs

Descriptive statistics and preliminary RIs of serum chemistry analytes for both shark species are presented in Table 1. Preliminary RIs with 90% CIs of the limits were calculated according to ASVCP recommendations for five serum chemistry analytes (glucose, triglycerides, total cholesterol, total protein and CK) in dusky sharks, the only species with the minimum required number of individuals (≥20) according to Friedrichs *et al.* (2012).

**Table 1:** Biochemical analyte data and preliminary RIs of wild dusky (*C. obscurus*) and sandbar (*C. plumbeus*) sharks from the eastern Mediterranean Sea in conventional units

**Analyte**	**Units**	**Dusky**										**Sandbar**					
		***n***	**Distribution**	**Descriptive statistics**				**RI with 90% CI**				***n***	**Distribution**	**Descriptive statistics**			
				**Mean**	**SD**	**Median**	**Range**	**LCI**	**RI**	**UCI**	**Method**			**Mean**	**SD**	**Median**	**Range**
Urea	mg/dL	15	G	2381	281	2351	2034–2971					12	G	2432	273	2390	2054–3043
Bilirubin	mg/dL	10	nG			0.16	0.01–0.95					8	G	0.1	0.05	0.06	0.01–0.13
Glucose	mg/dL	21	G	86	15	84	54–111	45–63	54–117	107–127	S	14	nG			65	46–94
Triglycerides	mg/dL	23	nG			47	7–148	0^‡^–9	0^‡^–207	143–274	RT	14	G	51	25	43	22–106
Cholesterol	mg/dL	23	G	82	38	74	28–156	0^‡^–33	23–179	145–218	ST	14	G	112	27	110	65–154
Total protein	g/dL	21	G	4.1	0.6	4.2	3.3–5.5	2.9–3.4	3.1–5.5	5.1–5.9	ST	12	G	4	0.3	3.9	3.6–4.5
Amylase	U/L	16	nG			64	1–375					9	nG			7	0.5–143
GGT	U/L	15	nG			28	3–224					9	nG			17	3–268
CK	U/L	21	nG			4589	537–34 048	414–720	444–32 569	18 455–55 027	RT	12	nG			7393	885–83 546
AST	U/L	17	nG			51	14–199					11	nG			51	21–179
K	mmol/L	18	G	4.6	0.7	4.6	3.2–6.2					11	G	4.3	1.1	4	2.7–6.1
P	mg/dL	17	nG			5.6	4.7–9.3					12	G	6.1	1.1	5.9	4.1–8
Cl	mmol/L	18	nG			302	252–320					12	G	298	14	295	282–329
Na	mmol/L	18	G	293	15	292	264–330					12	G	285	8	282	273–301
Ca	mg/dL	18	G	15.6	0.9	15.4	14.1–17.2					11	G	15.6	0.7	15.8	14.4–16.8

Abbreviations: N, number of individuals; LCI, lower 90% confidence limit; UCI, upper 90% confidence limit; S, standard method; ST, standard method after Box–Cox transformation; and RT, robust method after Box–Cox transformation. ^‡^Denotes a negative value that was replaced with zero.

**Figure 2:**
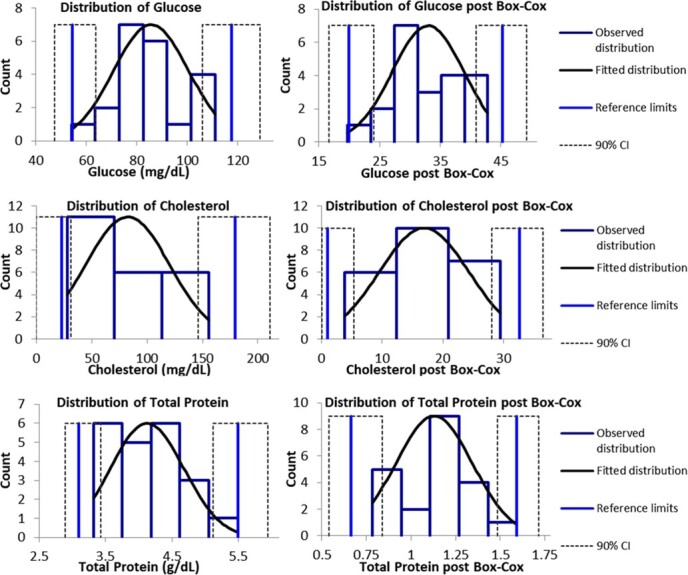
Distribution of glucose, cholesterol and total protein data before (left panels) and after (right panels) Box–Cox transformation with reference limits and 90% CIs for wild dusky (*C. obscurus*) sharks from the eastern Mediterranean Sea.

The robust method following Box–Cox transformation was used for analytes with non-Gaussian data, and the standard method was used for analytes with Gaussian data with or without Box–Cox transformation according to data distribution (Figure 2). Due to the small amount of data and its distribution, the software could not determine the lower limit of triglyceride RIs and their CI since they appeared as negative values and were therefore replaced with zero. This is also true for the CI of the lower limit of cholesterol values. The 90% CIs for glucose, total protein and CK exceeded the recommended 20%.

#### Serum chemistry analyte correlations with environmental parameters

Water temperature (°C) and salinity (ppt) data were obtained for 18 dusky sharks and all 14 sandbar sharks. The values attributed to each shark were measured at the specific drumline on which the sharks were caught or an average of all drumline measurements taken that day for sharks caught using handlines. Measured temperature and salinity did not differ between species capture events. Temperatures ranged between 18 and 26.9°C (mean ± SD, 22.8 ± 2°C), and salinities between 35.6 and 41 ppt (mean ± SD, 38.9 ± 1.4 ppt). In dusky sharks, salinity and temperature data correlated positively with serum sodium concentrations. Salinity also correlated positively with serum CK activities, and temperature correlated positively with serum chloride and calcium concentrations. In sandbar sharks, salinity correlated positively with serum calcium concentrations and negatively with serum CK, GGT and amylase activities, and triglyceride and urea concentrations. No correlations between temperature and serum chemistry analyte results for sandbar sharks were observed (Table 2).

**Table 2:** Statistically significant (*P* ≤ 0.05) Spearman's rank correlations between salinity and temperature data and biochemical analytes of wild dusky (*C. obscurus*) and sandbar (*C. plumbeus*) sharks from the eastern Mediterranean Sea

	**Dusky**				**Sandbar**	
**Analyte**	**Salinity**		**Temperature**		**Salinity**	
	***ρ***	***P***	***ρ***	***P***	***ρ***	***P***
Na	0.6	0.01	0.543	0.045		
Cl			0.588	0.027		
Ca			0.571	0.033	0.661	0.03
CK	0.59	0.01			−0.7	0.01
Urea					−0.63	0.03
Triglycerides					−0.59	0.03
GGT					−0.84	0.01
Amylase					−0.87	0

#### Serum chemistry analyte correlations with time of day and season

**Figure 3:**
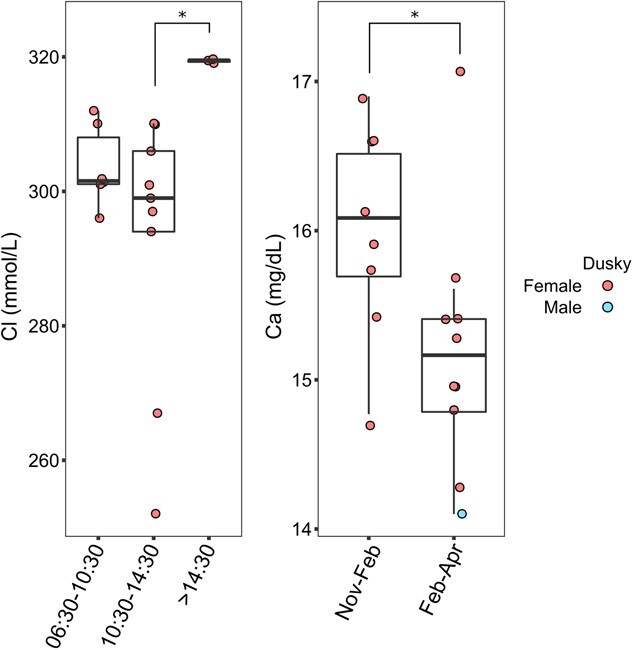
Chloride and calcium data of wild dusky (*C. obscurus*) sharks from the eastern Mediterranean Sea in partition according to time in the day and time in the season in which they were caught and sampled. Morning, *n* = 5; noon, *n* = 10; afternoon, *n* = 3. Beginning, *n* = 8; end, *n* = 10. ^*^Statistically significant (*P* ≤ 0.05) difference between groups.

In dusky sharks, serum chloride concentrations were higher (*χ*^2^ = 8.302, *df* = 2, *P*-adjusted = 0.012) in individuals captured between 14:30 and 18:00 h than in those captured between 10:30 and 14:30 h. Serum calcium concentrations were higher (*P* = 0.041) in sharks captured at the beginning of the sampling season (November–January) than in those caught towards its ending (February–April) (Figure 3).

#### Serum chemistry analyte associations with morphometric measurements and interspecific differences

**Figure 4:**
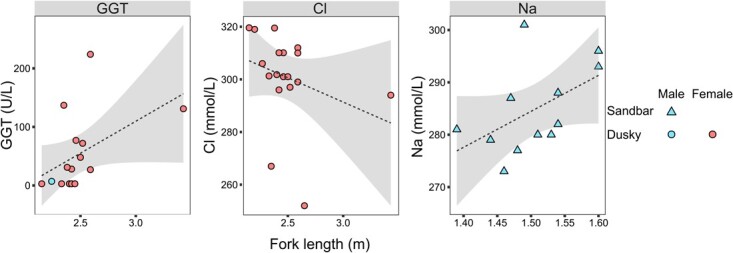
Statistically significant (*P* ≤ 0.05) Spearman's rank correlations between FL data and serum chemistry analytes of wild dusky (*C. obscurus*) and sandbar (*C. plumbeus*) sharks from the eastern Mediterranean Sea. The dashed line represents the trend line and the grey area the 95% CI of the trend line.

In dusky sharks, FL correlated positively with GGT activity (*ρ* = 0.534, *P* = 0.04) and negatively with chloride concentration (*ρ* = −0.483, *P* = 0.042). Sandbar shark FLs were positively correlated with sodium concentrations (*ρ* = 0.583, *P* = 0.046) (Figure 4).

**Figure 5:**
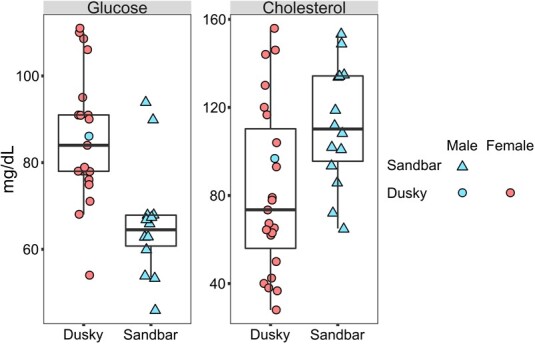
Glucose and cholesterol data of wild dusky (*C. obscurus*) and sandbar (*C. plumbeus*) sharks from the eastern Mediterranean Sea.

In the present study, statistically significant differences between dusky and sandbar sharks were only detected for two analytes, with dusky sharks exhibiting higher glucose (*W* = 255, *P* < 0.001) and lower total cholesterol (*t* = −2.515, *P* = 0.017) concentrations compared to sandbar sharks (Figure 5). Comparisons of serum chemistry values measured for the two shark species in the current study to other conspecific populations and other shark species in other locations are presented in Table 3. Detailed results of these comparisons are provided in Supplementary Table S2.

#### Serum chemistry analyte correlations with hook time

**Figure 6:**
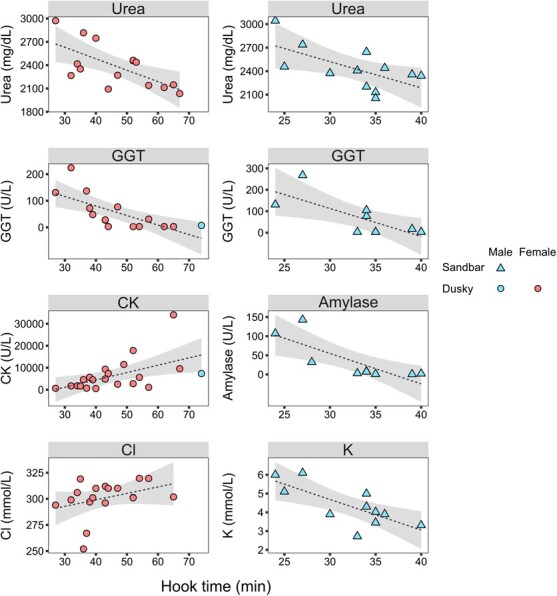
Statistically significant (*P* ≤ 0.05) Spearman's rank correlations between hook time data and serum chemistry analytes of wild dusky (*C. obscurus*) and sandbar (*C. plumbeus*) sharks from the eastern Mediterranean Sea. The dashed line represents the trend line and the grey area the 95% CI of the trend line.

Hook time in both species was correlated negatively with serum urea concentrations (dusky, *ρ* = −0.556, *P* = 0.031; sandbar: *ρ* = −0.646, *P* = 0.023) and GGT activity (dusky, *ρ* = −0.763, *P* < 0.001; sandbar, *ρ* = −0.667, *P* = 0.049). In dusky sharks, hook time correlated positively with serum chloride concentrations (*ρ* = 0.498, *P* = 0.035) and CK activity (*ρ* = −0.594, *P* = 0.005). In sandbar sharks, hook time correlated negatively with serum potassium concentrations (*ρ* = −0.65, *P* = 0.03) and amylase activities (*ρ* = −0.879, *P* = 0.002) (Figure 6).

### Haematology results

**Figure 7:**
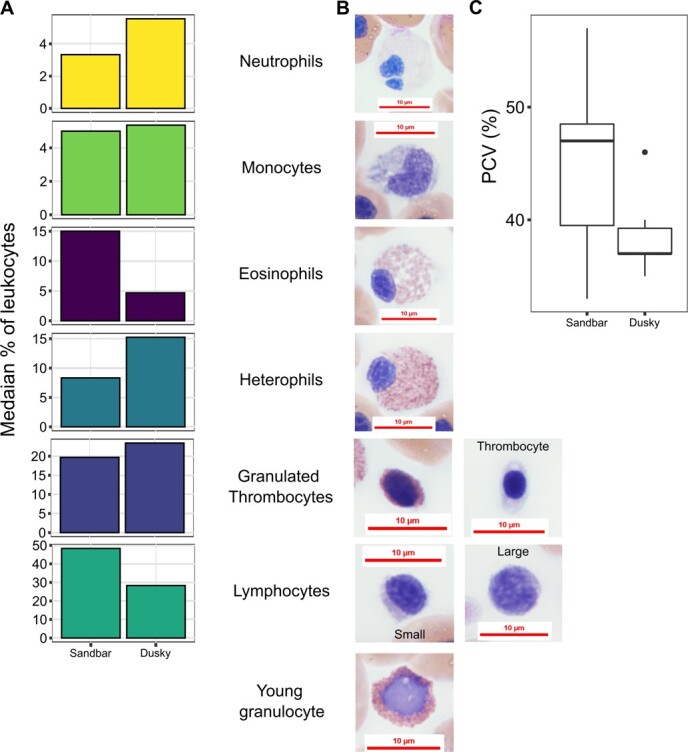
Haematological data of wild dusky (*C. obscurus*) and sandbar (*C. plumbeus*) sharks from the eastern Mediterranean Sea. (A) Results of leukocyte differential counts, *n* = 7 for both species; (B) images at ×100 magnification of Romanowsky-stained peripheral blood cells of dusky sharks caught during this study; (C) PCV data of the two species: dusky, *n* = 6; sandbar, *n* = 7.

**Figure 8:**
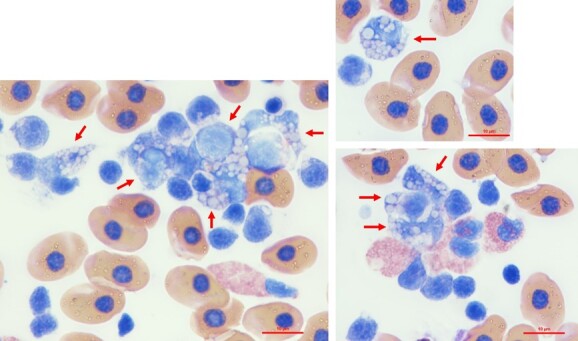
Images at ×100 magnification of Romanowsky-stained atypical monocytes (indicated by red arrows) of a dusky (*C. obscurus*) female sampled during this study.

Haematology data were composed of differential leukocyte counts and PCV measurements. These data were obtained for 14 of the sampled sharks: seven dusky sharks (females, 6; males, 1) and seven sandbar sharks (all males) (Figure 7). Detailed haematology data are presented in Supplementary Table S3. Blood cells were identified based on previous descriptions of sandbar shark blood cells (Haines and Arnold, 2014). Leukocytes were classified accordingly as neutrophils, monocytes, eosinophils, heterophils, lymphocytes and granulated thrombocytes for both species. In one dusky shark, multiple previously undescribed leukocytes were noted, presenting a dark blue cytoplasm and containing round inclusions of variable size, possibly composed of an unidentified phagocytized substance. These were interpreted as ‘atypical’ (highly vacuolated) monocytes (Figure 8).

## Discussion

The present study provides a novel report on serum and haematological data for free-ranging sandbar and dusky sharks from the eastern Mediterranean Sea. Preliminary RIs were proposed for five serum chemistry analytes (glucose, triglycerides, total cholesterol, total protein and CK) in the dusky sharks that reached the minimum sample size of 20 individuals. Serum sodium, chloride and calcium concentrations showed a positive relation to temperature and salinity conditions. Serum chloride and calcium concentrations showed varying temporal trends on a diurnal and seasonal scale. Serum sodium and chloride concentrations and CK activities showed varying relations to shark body size. Serum glucose and total cholesterol concentrations differed between the two shark groups (female dusky and male sandbar sharks). Comparisons were also made to reported serum chemistry values for conspecifics and other shark species elsewhere. Serum urea, chloride and potassium concentrations and CK, GGT and amylase activities showed varying relations to shark hook time. Both species' blood cell morphology and haematological data were also examined, though for a small sample size. Additionally, atypical, previously undescribed leukocytes, interpreted as monocytes, were noted in one dusky shark.

**Table 3:** Comparison of this study's results for wild dusky (*C. obscurus*) and sandbar (*C. plumbeus*) sharks from the eastern Mediterranean Sea with published biochemical analytes in conspecifics and other shark species elsewhere

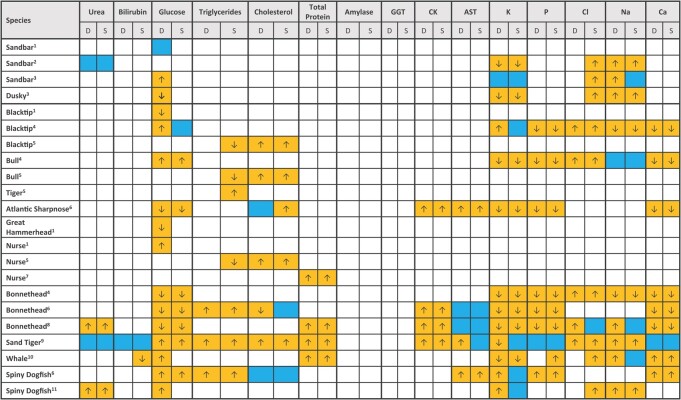

D, dusky shark; S, sandbar shark.

Blue colour denotes no significant difference between our data and the published value.

Orange colour denotes a significant difference between our data and the published value.

↑ Denotes our results are higher than the published value.

↓ Denotes our results are lower than the published value.

^a^ Jerome *et al.* (2017), ^b^Brill *et al.* (2008), ^c^Marshall *et al.* (2012), ^d^Manire *et al.* (2001), ^e^Gallagher *et al.* (2017), ^f^Haman *et al.* (2012), ^g^AtallahBenson *et al.* (2020), ^h^Harms *et al.* (2002), ^i^Otway (2015), ^j^Dove *et al.* (2010), ^k^Mandelman and Farrington (2007).

Sandbar (*C. plumbeus*); Dusky (*C. obscurus*); Blacktip (*Carcharhinus limbatus*); Bull (*C. leucas*); Tiger (*Galeocerdo cuvier*); Atlantic Sharpnose (*Rhizoprionodon terraenovae*); Great Hammerhead (*Sphyrna mokarran*); Nurse (*Ginglymostoma cirratum*); Bonnethead (*S. tiburo*); Sand Tiger (*C. taurus*); Whale (*R. typus*); Spiny Dogfish (*S. acanthias*).

### Serum chemistry interanalyte associations

Although the physiological function of amylase in sharks remains unclear, it is plausible that it is nutrition related (Hidalgo *et al.,* 1999). Therefore, the positive correlation noted between amylase activities and triglyceride concentrations in both shark species may reflect a postprandial association. GGT is a membrane-bound enzyme whose serum activity is mostly of a hepatocyte and biliary cell origin. Elevated serum GGT activity is a marker of cholestasis, which may also cause increased serum total cholesterol concentrations (Lawrence and Steiner, 2017). The reasons underlying the positive association between GGT activity and triglyceride concentrations noted in both shark species remain unclear and require further research. AST is a relatively nonspecific enzyme present in many soft tissues in mammals, but its concentrations are usually high in the skeletal muscles of many vertebrates. Hence, increased AST activity, as noted herein, is likely associated with capture and restraint-associated muscle damage (Otway *et al.,* 2011). Increased serum potassium, mostly an intracellular cation, has also been previously reported in elasmobranchs in relation to stress (Skomal and Mandelman, 2012). Therefore, the positive correlation detected between AST activities and potassium concentrations in both shark species may be attributed to both muscle cell damage and stress. Positive sodium correlations to chloride and calcium were observed for both species, likely due to their role as osmolytes, exhibiting a supportive role for urea and Trimethylamine N-oxide in ureotelic species, such as sharks (Ballantyne, 2016). Increases in serum CK and GGT activities can also be indicative of both skeletal muscle and hepatic impairment in vertebrates (Boyd, 1962). In fact, recent studies suggest CK and GGT be used with other hepatic markers to elucidate whether damages originate from skeletal muscles or the liver (Rosales *et al.,* 2008; Wosnick *et al.,* 2020). As the results of this study indicate that the increase in CK and GGT activities are not concomitant in dusky sharks, it is plausible to infer that organs other than the liver that also express GGT (*i.e.* kidneys, spleen) may be the source of increased activity in this species. As for sandbar sharks, the positive correlation detected between these analytes seems to indicate that both skeletal muscles and the liver are synthetizing and expressing such analytes, possibly due to extenuating exercise imposed by capture.

### Serum chemistry analyte descriptive statistics and preliminary RIs

In the study herein, even though dusky sharks are almost all females and sandbar sharks are all males, considerably limiting RI calculations, we decided to calculate preliminary RIs with 90% CIs of the limits for the only five serum analytes in dusky sharks that reached the required minimum sample size. Of course, the potential influence of capture-related stress on analyte concentrations and activities in the process of RIs determination for free-living sharks must be considered (Otway, 2015), as well as the fact that almost only females (all but one) were used for the assessment at the minimum recommended sample size. Furthermore, the particularities of the sampling site, mainly the presence of the coastal power and desalination plants, leading to warmer and more saline waters than in other areas and potential physiological alterations, should also be considered. Further studies should perform this calculation for males following the same sampling method and in the same population to establish baseline data for this group.

### Serum chemistry analyte correlations with environmental parameters

The study site where the investigated sharks were sampled is characterized by high water temperature and salinity caused by the outflow of the cooling water and brine from the onshore power and desalination plants. The outflow water is warmer by approximately 10°C and 1.9-fold more saline than the intake water. The warm and saline water plume extends southwest of the outflow. Bottom water at the study site is elevated by approximately 4°C and 5% salinity relative to the surrounding water. Temperature and salinity are presumably positively correlated at this study site (Cohen *et al.,* 2020). However, no significant correlation was found between these factors in our data, possibly due to the small sample size.

The positive relationship between salinity and sodium levels observed in the examined dusky sharks was expected due to the osmoregulatory function of this electrolyte. Calcium levels have also been shown to rise concomitantly with increasing salinity in bonnethead sharks (S*phyrna tiburo*) (Mandrup-Poulsen, 1981), similar to the trend observed herein for sandbar sharks. In ectothermic sharks, such as the two species examined in this study, haemoglobin–oxygen affinity decreases with temperature rise, and elevated blood oxygen partial pressure has been reported in juvenile sandbar sharks subjected to increased temperatures (Morrison *et al.,* 2015). As a result, the anaerobic metabolism is enhanced during exhaustive exercise under increased temperatures, leading to intensified acidosis. In response, sodium and chloride plasma concentrations increase through branchial ion exchange to maintain acid–base balance. However, the negative correlation observed in the examined sandbar sharks between salinity and urea, a pivotal osmolyte in elasmobranchs, remains unexplained. CK activity presented opposite trends associated with salinity in the two examined species, potentially coincidentally. Additionally, the inverse relation between salinity and the metabolic indices triglycerides, GGT and amylase noted in the examined sandbar sharks is also unexplained, and the mechanism behind it remains to be determined. However, it is important to note that this specific shark population is exposed to differential environmental conditions (warmer and more saline waters due to the proximity to the power and desalination plants) than expected for other populations, which should be considered in future assessments and comparisons to other populations.

### Serum chemistry analyte correlations with time of day and season

In dusky sharks, chloride levels were higher in individuals caught in the afternoon (14:30–18:00 h) than in those caught at noon (10:30–14:30 h), and calcium levels were higher in individuals caught at the beginning of the tagging season (November–January) than in those caught towards its end (February–April). These differences, however, cannot be explained based on the literature, and it is necessary to note that the sample sizes of the different groups were small. Thus, further assessments in this regard are required.

### Serum chemistry analyte associations with morphometric measurements and differences between species

In the dusky sharks examined herein, FL correlated positively with GGT and negatively with chloride, and in sandbar sharks, it correlated positively with sodium. The body sizes inferred from FL measurements of the dusky sharks in this study were more variable than those of the examined sandbar sharks, which were similar. As GGT plays a role in protein intake in intestinal cells (Lemieux *et al.,* 1999), it is plausible that the larger dusky sharks consume larger prey with higher protein content and require more GGT for its absorption. Ferguson *et al.* (1993) reported that exhaustive exercise induces higher white muscle cell acidosis in larger vs. smaller fish in rainbow trout (*Oncorhynchus mykiss*), with higher numbers of metabolic protons produced by larger fish than by smaller ones. Chloride and sodium are tightly linked to acid–base dynamics and branchial ion exchange. During recovery from acidemia, active branchial exchange of H^+^ ions induce a cation influx from the environment, including sodium, leading to increased plasma concentrations (Skomal and Mandelman, 2012). It is plausible that the positive correlation between body size and sodium levels observed herein in sandbar sharks is a result of this process. Brill *et al.* (2008) observed red blood cell (RBC) swelling in juvenile sandbar sharks following anaerobic exercise, suggesting that the mechanism leading to this swelling comprises a decrease in the intracellular net negative charge that, in turn, leads to an influx of chloride ions from the plasma to preserve intracellular electroneutrality, followed by an influx of water and cell swelling. Further studies are required to elucidate if a similar mechanism could underlie the inverse relationship between body size and chloride levels observed herein in dusky sharks. Moreover, as sharks of different sizes can move across salinity gradients (Pillans and Franklin, 2004), future studies should also consider the role of salinity in chloride and sodium dynamics in relation to body size.

All but one dusky shark were female and all sandbar sharks were male, and, although all sharks were adults, the sampled dusky sharks were larger than the sandbar sharks. Moreover, hook times were longer for the sampled dusky sharks than those inflicted on the sandbar sharks. Glucose levels were higher in dusky sharks vs. sandbar sharks, and total cholesterol levels displayed the opposite association between the two species. No other statistical difference was found between the species for any analytes employed in the present study. Traditionally, glucose is used as a reliable secondary stress marker for vertebrates. However, recent studies indicate that this analyte may not be as efficient when analysed alone in the case of sharks. For instance, sharks rely on a diet rich in protein (>20% of the total nutritional composition) and fatty acids but low in carbohydrates (Speers-Roesch and Treberg, 2010). Such a nutritional composition already indicates that glucose will not be the predominant metabolic energy source, with ketone bodies comprising the primary fuel in response to acute stress. Moreover, sharks display a very distinct metabolism compared to other vertebrates. The fact that cortisol is not the glucocorticoid expressed by these animals makes glucose scarcely relevant to stress responses upon capture (Hoffmayer *et al.,* 2012). Nonetheless, glucose can still be used as an energy marker upon capture that can behave in two opposite ways: increasing or decreasing during fight-or-flight situations (Romero and Beattie, 2022), and both can be interpreted as adaptive responses to the metabolic demands necessary under stressful circumstances.

Elevated glucose levels, or hyperglycemia, have been documented in several shark species, including those investigated herein (Cliff and Thurman, 1984; Spargo, 2001). Stress recovery times necessary for hyperglycemia have been reported as over 24 hours for dusky sharks (Cliff and Thurman, 1984) and under 24 hours for sandbar sharks (Spargo, 2001). In a study by Marshall *et al.* (2012), glucose levels were measured in longline-captured sharks belonging to 11 species, including dusky and sandbar sharks. Sandbar glucose levels were significantly lower than all other examined shark species (including the dusky shark), except for two individuals. Morgan and Burgess (2007) reported relatively low at-vessel mortality rates for sandbar sharks (36.1% total, 22.4% adult) and high at-vessel mortality rates for dusky sharks (81.1% total, 44.4% adult). In the present study, the higher glucose levels observed in dusky sharks vs. sandbar sharks may have resulted from extended species-specific demands to overcome the allostatic overload imposed by capture. More specifically, more vulnerable shark species can deplete their primary energy reserves in stressful situations, requiring mobilization of other energy fuels (*e.g.* triglycerides and glucose). It is also possible that larger sharks exhibit alternative metabolic pathways to ketone body use to ensure adequate energy mobilization for daily activities and stressful situations. However, a diet/nutrient intake role cannot be ruled out, as differences in energy marker mobilization may simply be the result of the species' diet.

Concerning total cholesterol, higher concentrations were detected in sandbar sharks compared to dusky sharks. Cholesterol plays a key role in male hormone production (Gao *et al.,* 2018) and is pivotal in ensuring sperm quality and protection (Beer-Ljubić *et al.,* 2009). Cholesterol is also important for cell membrane structuring and as a sterol carrier (Ritter and Dempsey, 1971; Yang *et al.,* 2016). Therefore, circulating cholesterol levels can also increase in the face of stressors due to cellular disruption and the need for rapid energy mobilization, respectively. Thus, it is possible to infer that the higher levels observed in the sandbar sharks are because they were all adult males, potentially close to their reproductive period. Alternatively, it is possible that the sandbar sharks were mobilizing cholesterol as an alternative stress response for the rapid mobilization of ketone bodies through the transport of the necessary substrates, which could explain why glucose levels were not as high as in dusky sharks.

Potassium levels in sharks from both species were in agreement with or lower than reported in other studies (Brill *et al.,* 2008; Marshall *et al.,* 2012). Hyperkalemia can result from kidney impairment, tissue damage and capture-related stress. In severe cases (potassium levels exceeding 7 mmol/L), it can cause tetany, disrupt myocardial function and possibly lead to death (Otway *et al.,* 2011). Chloride and sodium levels in both species examined herein were predominantly higher than reported for these species in other assessments (Brill *et al.,* 2008; Marshall *et al.,* 2012). Chloride and sodium are the main inorganic salts in fish blood and essential in maintaining blood electroneutrality. Although increases in chloride and/or sodium concentrations have been associated with capture stress in several elasmobranch species, others display no stress-related effect on these ions. Thus, stress effects on secondary osmotic properties in elasmobranchs are variable and unpredictable (Skomal and Mandelman, 2012). It is possible the high chloride and sodium concentrations determined herein were related to a compensatory role of both ions to a potential reduction in urea levels, acting as secondary buffers to ensure allostasis. However, it is also possible that higher levels result from differences at the population level, as the oceanographic/biochemical conditions of the studied area are distinct from other regions, posing additional challenges to shark osmoregulation. More specifically, the area where they aggregate in the winter is characterized by particularly high water temperatures and salinity due to the cooling water and brine discharged from the Hadera power and desalination plants (Cohen *et al.,* 2020). Warmer temperatures have been reported as altering shark physiology (Bockus *et al.,* 2020). That being said, as urea production and maintenance costs are very high, the sharks in this area may rely on secondary osmolytes to deal with local osmotic challenges. The urea levels measured in both species examined in this study were consistent with previously reported values for juvenile sandbar sharks (Brill *et al.,* 2008). Urea is one of the main elasmobranch osmolytes, and decreased urea levels can originate from liver (responsible for urea synthesis) or kidney (responsible for urea retention) impairment (Otway *et al.,* 2011) and, occasionally, stress (Skomal and Mandelman, 2012).

As vertebrate haematology and serum chemistry vary with species, geographic area, ecological habitat, genetically distinct populations, sex, life stage and more (Jensen *et al.,* 1994; Maceda-Veiga *et al.,* 2015; Page-Karjian *et al.,* 2015), it is not common to compare values measured in a specific population with those of different species in other areas. However, due to the paucity of published reference data for sharks, such comparisons were the only available. Even so, data were scarce, and no reference was found for GGT values in any shark species. Furthermore, in the only previously reported study of amylase activity in sharks (Haman *et al.,* 2012), the employed assay (dry chemistry; Ortho Vitros, Rochester, NY) was different than the present method (wet chemistry) using different reagents and reaction conditions, and hence, direct comparison cannot be performed. Additionally, we could not statistically compare our non-normally distributed analyte results to published values reported as mean ± SD. These comparison-restricting factors should be considered when examining similarities and differences between the findings reported herein and those reported by others and speculating regarding their origin.

Both species analysed herein displayed higher total protein and CK levels than reported in the literature for other shark species, with CK levels higher by 1–2 orders of magnitude than others'. The extremely high CK activities determined herein are likely attributed to leakage of this intracellular enzyme from muscle cells due to impaired cell membrane or cell disruption. This is caused by metabolic and respiratory cell acidosis following exhaustive activity during capture struggle (Cliff and Thurman, 1984). However, high CK activity can also indicate exhaustive exercise with no further debt to the systemic health of evaluated sharks. For example, Brill *et al.* (2008) reported a ~16% increase in plasma protein in juvenile sandbar sharks postanaerobic exercise accompanying catch-and-release fishing. They associated this result with the fact that plasma proteins can account for over 70% of whole blood pH buffering capacity in elasmobranchs. Alternatively, the interspecific difference in total protein levels between the two species examined herein and other shark species described in the literature may be attributed to dietary differences (Manire *et al.,* 2001). AST activities of the two examined species were similar to or higher than the values reported for other shark species (Harms *et al.,* 2002; Haman *et al.,* 2012 ; Otway, 2015). Increased AST activities can result from liver dysfunction, skeletal or cardiac muscle impairment, septicemia or capture-related stress (Otway *et al.,* 2011). In this regard, Manire *et al.* (2001) examined serological changes associated with gill-net capture and restraint in three shark species. They found elevated AST levels only in one, the bull shark (*Carcharhinus leucas*). The authors attributed this result to cell damage caused by capture struggle, which was much shorter in the other two examined species. Triglyceride levels measured in this study in sandbar sharks were significantly higher than reported for other shark species (Haman *et al.,* 2012; Otway, 2015). Triglycerides are a lipid class that plays an essential role in energy transport and is frequently analysed in metabolism studies. These compounds are a primary source of metabolic energy in numerous organisms and are considered quick to respond to feeding changes (Gallagher *et al.,* 2017). The high triglyceride levels observed in the sandbar sharks in this study are possibly postprandial, resulting from recent predation. Bilirubin levels in both species were similar to the values reported for sand tiger sharks (*Carcharias taurus*) (Otway, 2015), and sandbar levels were higher than the values reported for whale sharks (*Rhincodon typus*) from the Georgia Aquarium (Dove *et al.,* 2010). Bilirubin is the product of the heme catabolism that is transported to the liver and, from there, to the bile and serves as a bile component in lipid digestion in the intestine (Lawrence and Steiner, 2017). In healthy animals, it is excreted by hepatocytes at a constant rate, and elevated plasma levels can indicate several liver dysfunctions (Wells *et al.,* 1986). Increased bilirubin levels in elasmobranchs can be the result of impaired uptake, conjugation or excretion in hepatocytes, damaged or obstructed bile ducts causing bile accumulation or prolonged fasting and confinement (Wosnick *et al.,* 2020). It is plausible that the lower bilirubin levels observed in the two captive whale sharks studied by Dove *et al.* (2010) relative to the other species are related to the different lipid content of their planktivorous diet, in contrast to the piscivorous diet of dusky, sandbar, and sand tiger sharks. However, the very small sample size and the effects of artificial feeding in captivity on whale sharks should also be considered. Thus, the levels observed in the present study indicate adequate functioning of the hepatobiliary system and satisfactory feeding intervals. Finally, the phosphorus and calcium concentrations determined in this study for both examined species were mostly lower than reported in the literature for other shark species (Manire *et al.,* 2001; Harms *et al.,* 2002; Haman *et al.,* 2012), although some reports were similar to (Otway, 2015) or lower than (Dove *et al.,* 2010; Haman *et al.,* 2012) our results.

### Serum chemistry analyte correlations with hook time

Hook time (from capture to release) in both species examined herein was negatively correlated with urea concentrations and GGT activities. In dusky sharks, hook time also correlated positively with CK activities and chloride concentrations, while in sandbar sharks, hook time correlated negatively with amylase activities and potassium concentration. Several shark stress studies reported a stress-related decrease in plasma urea concentration, attributed to urea loss through stress-induced increased branchial urea permeability (Skomal and Mandelman, 2012). Another physiological stress manifestation is inhibition of the gastrointestinal system at the gastric transit and motility levels (Chrousos, 2009). Therefore, it is possible that the negative correlation between hook time and the digestive enzymes GGT and amylase is due to digestive activity inhibition during stress. Similarly, Liu *et al.* (2018) reported that Nile tilapia (*Oreochromis niloticus*) fingerling stress caused by high stocking density leads to depressed amylase and other digestive enzyme activities. Exhaustive activity during capture struggle causes cell acidosis resulting in increased permeability of cell membrane and cell disruption. Thus, the intracellular enzyme CK leaks to the plasma (Cliff and Thurman, 1984), and its plasma concentrations build up with prolonged struggle. Elevated serum chloride levels were reported by Mandelman and Farrington (2007) in spiny dogfish (S*qualus acanthias*) post-otter-trawl capture, decreasing over the following transport (2 hours) and captivity (30 days) periods. Stress-induced increases in serum sodium and/or chloride have been documented in some elasmobranchs, although other studies reported unchanged concentrations following stress exposure. Further investigations are required to decipher the mechanisms controlling the presence/absence of these trends so that sodium and chloride may serve as reliable indicators of stress in elasmobranchs (Skomal and Mandelman, 2012). The inverse relationship between hook time and potassium levels observed in the examined sandbar sharks stands in contrast to the findings of several elasmobranch stress studies that report marked plasma potassium increase (Wells *et al.,* 1986; Manire *et al.,* 2001; Mandelman and Farrington, 2007), although Brill *et al.* (2008) noted a 13% decrease in serum potassium concentrations in juvenile sandbar sharks following anaerobic exercise. Therefore, the link between potassium levels and stress in sharks remains to be elucidated by future studies.

### Haematology

The current results of the differential leukocyte counts in the sandbar sharks were in agreement with the values reported for this species by Arnold (2005), while the results for the dusky sharks differed slightly, namely for lymphocyte, heterophil, and eosinophil percentages.

In addition, the two groups differ in sex. Similarly to the results reported herein, Persky *et al.* (2012) reported significantly higher heterophil percentages in female vs. male smooth dogfish (*Mustelus canis*). Furthermore, Persky *et al.* (2012) reported significantly higher PCV values in male vs. female smooth dogfish. In contrast, the PCV measurements of the present study in both examined species were substantially higher than the values reported in the literature, which also varies among studies (Emery, 1986; Arnold, 2005; Brill *et al.,* 2008; Marshall *et al.,* 2012; Jerome *et al.,* 2017). This is most likely due to variability in capillary centrifugation time and force, which were relatively low in the current study (2570*g* for 3 minutes and 20 seconds vs. 11 000*g* for 5 minutes recommended by Arnold, 2005). Although the observed PCV values showed no statistically significant difference between the two examined species, the sandbar values displayed a much wider range due to higher maximal values. Elevated PCV has been previously documented in juvenile sandbar sharks subjected to anaerobic exercise through RBC swelling following cell acidosis (Brill *et al.,* 2008). However, this phenomenon has yet to be studied in the dusky shark. Marshall *et al.* (2012), who also examined both species, observed higher PCV values in sandbar sharks compared to dusky sharks, while Emery (1986) reported the opposite.

To our knowledge, atypical monocytes such as those observed in one of the dusky specimens in the current study have not been documented in sharks. There appears to be some phagocytosis of these cells, which is nonspecific and can be seen with concurrent inflammatory leukogram but unknown significance. This is often seen in nonmammalian species during or after rehabilitation (Dr Nicole I. Stacy, personal communication). The specimen with the atypical monocytes also presented relatively high percentages of eosinophils and granulated thrombocytes. Further assessments in this regard should be conducted due to the low sample size.

## Conclusions

The data reported herein establish the first database on dusky and sandbar shark serum chemistry and haematology in the eastern Mediterranean Sea. The determined associations between serum chemistry analytes and stress, body size and environmental conditions indicate interspecies differences probably related to physiological aspects and distinct metabolic pathways. Exploratory RIs were calculated for some analytes in dusky sharks, although these data are still preliminary and should be viewed with caution. Understanding how physiological variables relate to abiotic factors and anthropogenic stressors is extremely important for conservation planning, especially for taxonomic groups as threatened as sharks. First, the collected data and correlations detected between markers and body condition scores allowed for the establishment of initial data on the health condition of the studied populations, providing basis for long-term monitoring. Second, the chosen markers and their relationship with hook times generate unprecedented data for the region, which, despite being a no-take zone for sharks, still suffers from bycatch. Thus, the collected data aid in management measures to reduce the impacts of incidental shark captures. In addition, the obtained data may improve nonlethal scientific captures, aiming to further reduce the stress imposed on the studied sharks. Third, data correlating shark physiology with environmental variables are scarce, configuring a knowledge gap in a changing world and its environmental consequences. Thus, the data generated in the present study contribute significantly to shark ecophysiology, which in turn can aid in conservation measures based on species resilience and overall population health and fitness. Additionally, the particularities of the study area (hot saline water due to the power and desalination plants) allow for unprecedented physiological data, as the studied sharks are exposed to very distinct conditions still little explored for most shark species, especially those large sized. Moreover, the data obtained in the present study will be instrumental in future assessments aiming to understand the particularities of the Mediterranean population that has been suffering very expressive declines for decades. It will serve studies focused not only in the region but also in comparative investigations between different populations, as the resilience of a species or an entire population is strongly mediated by physiological factors and the ability to adapt to different conditions and stressors.

## Funding

This study was financially supported by the Kahn Foundation and by the Hong Kong Branch of Southern Marine Science and Engineering Guangdong Laboratory (Guangzhou, China) (SMSEGL20SC02).

## Data Availability Statement

The datasets generated for this study are available on request to the corresponding author.

## Author contributions

D.M., A.S., D.T., I.A. and N.W. conceived the presented idea. D.M., A.S., D.T., N.D., T.S.M. and N.W. developed the theory. T.S.M. performed the computations. D.M., I.A., E.B., L.L. and N.W. verified the analytical methods. R.A.H.D. and N.W. evaluated and discussed the data. D.M., A.S., D.T. and I.A. supervised the findings of this work. All authors discussed the results and contributed to the final manuscript.

## Supplementary Material

Web_Material_coad037

## References

[ref1] Abdul Malak D , Livingstone SR, Pollard D, Polidoro BA, Cuttelod A, Bariche M et al. (2011) Overview of the Conservation Status of the Marine Fishes of the Mediterranean Sea. IUCN, Gland Switzerland, and Malaga, Spain

[ref2] Arnold JE (1997) Preliminary hematology study of the sandbar shark, *Carcharhinus plumbeus*. In Proceedings: International Association of Aquatic Animal Medicine 28th Annual Conference (Harderwijk, the Netherlands), pp. 53–54

[ref3] Arnold JE (2005) Hematology of the sandbar shark, *Carcharhinus plumbeus*: standardization of complete blood count techniques for elasmobranchs. Vet Clin Pathol 34: 115–123. 10.1111/j.1939-165X.2005.tb00023.x. 15902662

[ref72] AtallahBenson L , Merly L, Cray C, Hammerschlag N (2020) Serum protein analysis of nurse sharks. J Aquat Anim Health 32: 77–82. 10.1002/aah.10100. 32012365

[ref4] Ballantyne JS (2016) Some of the most interesting things we know, and don't know, about the biochemistry and physiology of elasmobranch fishes (sharks, skates and rays). Comp Biochem Physiol B Biochem Mol Biol 199: 21–28. 10.1016/j.cbpb.2016.03.005. 26969804

[ref5] Barash A , Pickholtz R, Pickholtz E, Blaustein L, Rilov G (2018) Seasonal aggregations of sharks near coastal power plants in Israel: an emerging phenomenon. Mar Ecol Prog Ser 590: 145–154. 10.3354/meps12478.

[ref6] Beer-Ljubić B , Aladrović J, Marenjak TS, Laškaj R, Majić-Balić I, Milinković-Tur S (2009) Cholesterol concentration in seminal plasma as a predictive tool for quality semen evaluation. Theriogenology 72: 1132–1140. 10.1016/j.theriogenology.2009.07.009. 19767087

[ref7] Bockus AB , LaBreck CJ, Camberg JL, Collie JS, Seibel BA (2020) Thermal range and physiological tolerance mechanisms in two shark species from the Northwest Atlantic. Biol Bull Rev 238: 131–144. 10.1086/708718. 32412839

[ref8] Bouyoucos IA , Talwar BS, Brooks EJ, Brownscombe JW, Cooke SJ, Suski CD, Mandelman JW (2018) Exercise intensity while hooked is associated with physiological status of longline-captured sharks. Conserv Physiol 6: coy074. 10.1093/conphys/coy074. 30591841 PMC6301290

[ref9] Boyd JW (1962) The comparative activity of some enzymes in sheep, cattle and rats—normal serum and tissue levels and changes during experimental liver necrosis. Res Vet Sci 3: 256–270. 10.1016/S0034-5288(18)34899-9.

[ref10] Brill R , Bushnell P, Schroff S, Seifert R, Galvin M (2008) Effects of anaerobic exercise accompanying catch-and-release fishing on blood-oxygen affinity of the sandbar shark (*Carcharhinus plumbeus*, Nardo). J Exp Mar Biol Ecol 354: 132–143. 10.1016/j.jembe.2007.10.011.

[ref11] Cavanagh RD , Gibson C (2007) Overview of the Conservation Status of Cartilaginous Fishes (Chondrichthyans) in the Mediterranean Sea. IUCN, Gland, Switzerland, and Malaga, Spain

[ref12] Chrousos GP (2009) Stress and disorders of the stress system. Nat Rev Endocrinol 5: 374–381. 10.1038/nrendo.2009.106. 19488073

[ref13] Cliff G , Thurman GD (1984) Pathological and physiological effects of stress during capture and transport in the juvenile dusky shark, *Carcharhinus obscurus*. Comp Biochem Physiol A Mol Integr Physiol 78: 167–173. 10.1016/0300-9629(84)90111-7.

[ref14] Cohen Y , Avramzon K, Levy G, Scwartz H (2020) Marine and coastal environmental monitoring report for 2019—Orot Rabin power station and Hadera desalination plant (H2ID).

[ref15] Coll M , Navarro J, Palomera I (2013) Ecological role, fishing impact, and management options for the recovery of a Mediterranean endemic skate by means of food. Biol Conserv 157: 108–120. 10.1016/j.biocon.2012.06.029.

[ref16] Coll M , Piroddi C, Steenbeek J, Kaschner K, Ben Rais Lasram F, Aguzzi J, Ballesteros E, Bianchi CN, Corbera J, Dailianis T et al. (2010) The biodiversity of the Mediterranean Sea: estimates, patterns, and threats. PLoS One 5: e11842. 10.1371/journal.pone.0011842. 20689844 PMC2914016

[ref17] Cortés E (2004) Life history patterns, demography, and population dynamics. In JC Carrier, JA Musick, eds, Biology of Sharks and Their Relatives. Heithaus MR, Boca Raton, FL, pp. 449–469

[ref18] Dove AD , Arnold J, Clauss TM (2010) Blood cells and serum chemistry in the world's largest fish: the whale shark *Rhincodon typus*. Aquat Biol 9: 177–183. 10.3354/ab00252.

[ref19] Dudley SFJ , Cliff G, Zungu MP, Smale MJ (2005) Sharks caught in the protective gill nets off KwaZulu-Natal, South Africa. 10. The dusky shark *Carcharhinus obscurus* (Lesueur 1818). Afr J Mar Sci 27: 107–127. 10.2989/18142320509504072.

[ref20] Dulvy NK , Fowler SL, Musick JA, Cavanagh RD, Kyne PM, Harrison LR, Carlson JK, Davidson LNK, Fordham SV, Francis MP et al. (2014) Extinction risk and conservation of the world's sharks and rays. Elife 3: e00590. 10.7554/eLife.00590. 24448405 PMC3897121

[ref21] Dulvy NK , Pacoureau N, Rigby CL, Pollom RA, Jabado RW, Ebert DA, Finucci B, Pollock CM, Cheok J, Derrick DH et al. (2021) Overfishing drives over one-third of all sharks and rays toward a global extinction crisis. Curr Biol 31: 4773–4787.e8. 10.1016/j.cub.2021.08.062. 34492229

[ref22] Edelist D , Rilov G (2014) Fishery trends in the Israeli Mediterranean. Ecol Environ 5: 90–97 (In Hebrew).

[ref23] Emery SH (1986) Hematological comparisons of endothermic vs ectothermic elasmobranch fishes. Copeia 1986: 700–705. 10.2307/1444952.

[ref24] Ferguson RA , Kieffer JD, Tufts BL (1993) The effects of body size on the acid-base and metabolite status in the white muscle of rainbow trout before and after exhaustive exercise. J Exp Biol 180: 195–207. 10.1242/jeb.180.1.195.

[ref25] Ferretti F , Myers RA, Serena F, Lotze HK (2008) Loss of large predatory sharks from the Mediterranean Sea. Conserv Biol 22: 952–964. 10.1111/j.1523-1739.2008.00938.x. 18544092

[ref26] Friedrichs KR , Harr KE, Freeman KP, Szladovits B, Walton RM, Barnhart KF, Blanco-Chavez J, American Society for Veterinary Clinical Pathology (2012) ASVCP reference interval guidelines: determination of de novo reference intervals in veterinary species and other related topics. Vet Clin Pathol 41: 441–453. 10.1111/vcp.12006. 23240820

[ref27] Gallagher AJ , Skubel RA, Pethybridge HR, Hammerschlag N (2017) Energy metabolism in mobile, wild-sampled sharks inferred by plasma lipids. Conserv Physiol 5: cox002. 10.1093/conphys/cox002. 28852506 PMC5570055

[ref28] Gao F , Li G, Liu C, Gao H, Wang H, Liu W, Chen M, Shang Y, Wang L, Shi J et al. (2018) Autophagy regulates testosterone synthesis by facilitating cholesterol uptake in Leydig cells. J Cell Biol 217: 2103–2119. 10.1083/jcb.201710078. 29618492 PMC5987723

[ref29] Geffré A , Concordet D, Braun JP, Trumel C (2011) Reference value advisor: a new freeware set of macroinstructions to calculate reference intervals with Microsoft excel. Vet Clin Pathol 40: 107–112. 10.1111/j.1939-165X.2011.00287.x. 21366659

[ref30] Haines AN , Arnold JE (2014) Elasmobranch blood cells. In SL Smith, RB Sim, MF Flajnik, eds, Immunobiology of the Shark. CRC Press, Boca Raton, FL, pp. 89–104

[ref31] Haman KH , Norton TM, Thomas AC, Dove ADM, Tseng F (2012) Baseline health parameters and species comparisons among free-ranging Atlantic sharpnose (*Rhizoprionodon terraenovae*), bonnethead (*Sphyrna tiburo*), and spiny dogfish (*Squalus acanthias*) sharks in Georgia, Florida, and Washington, USA. J Wildl Dis 48: 295–306. 10.7589/0090-3558-48.2.295. 22493105

[ref32] Harms C , Ross T, Segars A (2002) Plasma biochemistry of bonnethead sharks, *Sphyrna tiburo*. Vet Clin Pathol 31: 111–115. 10.1111/j.1939-165X.2002.tb00289.x. 12189596

[ref33] Hidalgo MC , Urea E, Sanz A (1999) Comparative study of digestive enzymes in fish with different nutritional habits. Proteolytic and amylase activities. Annu Rev Fish Dis 170: 267–283. 10.1016/S0044-8486(98)00413-X.

[ref34] Hoffmayer ER , Hendon JM, Parsons GR (2012) Seasonal modulation in the secondary stress response of a carcharhinid shark, *Rhizoprionodon terraenovae*. Comp Biochem Physiol A Mol Integr Physiol 162: 81–87. 10.1016/j.cbpa.2011.05.002. 21596154

[ref35] Horn PS , Pesce AJ, Copeland BE (1998) A robust approach to reference interval estimation and evaluation. Clin Chem 44: 622–631. 10.1093/clinchem/44.3.622. 9510871

[ref36] IUCN (2021) IUCN red list of threatened species. Version 2021-3 Available at: https://www.iucnredlist.org [Accessed May 6, 2022].

[ref37] Jensen AL , Wenck A, Koch J, Poulsen JSD (1994) Comparison of results of haematological and clinical chemical analyses of blood samples obtained from the cephalic and external jugular veins in dogs. Res Vet Sci 56: 24–29. 10.1016/0034-5288(94)90191-0. 8146449

[ref38] Jerome JM , Gallagher AJ, Cooke SJ, Hammerschlag N (2018) Integrating reflexes with physiological measures to evaluate coastal shark stress response to capture. ICES J Mar Sci 75: 796–804. 10.1093/icesjms/fsx191.

[ref39] Lawrence YA , Steiner JM (2017) Laboratory evaluation of the liver. Vet Clin North Am Small Anim Pract 47: 539–553. 10.1016/j.cvsm.2016.11.005. 28063744

[ref40] Lemieux H , Blier P, Dutil JD (1999) Do digestive enzymes set a physiological limit on growth rate and food conversion efficiency in the Atlantic cod (*Gadus morhua*)? Fish Physiol Biochem 20: 293–303. 10.1023/A:1007791019523.

[ref41] Liu G , Ye Z, Liu D, Zhao J, Sivaramasamy E, Deng Y et al. (2018) Influence of stocking density on growth, digestive enzyme activities, immune responses, antioxidant of *Oreochromis niloticus* fingerlings in biofloc systems. Fish Shellfish Immunol 81: 416–422. 10.1016/j.fsi.2018.07.047. 30056209

[ref42] Maceda-Veiga A , Figuerola J, Martínez-Silvestre A, Viscor G, Ferrari N, Pacheco M (2015) Inside the Redbox: applications of haematology in wildlife monitoring and ecosystem health assessment. Sci Total Environ 514: 322–332. 10.1016/j.scitotenv.2015.02.004. 25668285

[ref43] Mandelman JW , Farrington MA (2007) The physiological status and mortality associated with otter-trawl capture, transport, and captivity of an exploited elasmobranch, *Squalus acanthias*. ICES J Mar Sci 64: 122–130. 10.1093/icesjms/fsl003.

[ref44] Mandrup-Poulsen J (1981) Changes in selected blood serum constituents, as a function of salinity variations, in the marine elasmobranch, *Sphyrna tiburo*. Comp Biochem Physiol A Mol Integr Physiol 70: 127–131. 10.1016/0300-9629(81)90408-4.

[ref45] Manire CA , Hueter RE, Hull E, Spieler R (2001) Serological changes associated with gill-net capture and restraint in three species of sharks. Trans Am Fish Soc 130: 1038–1048. 10.1577/1548-8659(2001)130<1038:SCAWGN>2.0.CO;2.

[ref46] Marshall H , Field L, Afiadata A, Sepulveda C, Skomal G, Bernal D (2012) Hematological indicators of stress in longline-captured sharks. Comp Biochem Physiol A Mol Integr Physiol 162: 121–129. 10.1016/j.cbpa.2012.02.008. 22353217

[ref47] Morgan A , Burgess GH (2007) At-vessel fishing mortality for six species of sharks caught in the Northwest Atlantic and Gulf of Mexico. Gulf Caribb Res 19: 123–129. 10.18785/gcr.1902.15.

[ref48] Morrison PR , Gilmour KM, Brauner CJ (2015) Oxygen and carbon dioxide transport in elasmobranchs. In RE Shadwick, AP Farrell, CJ Brauner, eds, Fish Physiol. Elsevier Inc, Lon don, San Diego, Oxford, pp. 127–219

[ref49] Otway NM (2015) Serum biochemical reference intervals for free-living sand tiger sharks (*Carcharias taurus*) from east Australian waters. Vet Clin Pathol 44: 262–274. 10.1111/vcp.12254. 25865808

[ref50] Otway NM , Ellis MT, Starr R (2011) Serum biochemical reference intervals for wild dwarf ornate wobbegong sharks (*Orectolobus ornatus*). Vet Clin Pathol 40: 361–367. 10.1111/j.1939-165X.2011.00330.x. 21790697

[ref51] Pacoureau N , Rigby CL, Kyne PM, Sherley RB, Winker H, Carlson JK, Fordham SV, Barreto R, Fernando D, Francis MP et al. (2021) Half a century of global decline in oceanic sharks and rays. Nature 589: 567–571. 10.1038/s41586-020-03173-9. 33505035

[ref52] Page-Karjian A , Rivera S, Torres F, Diez C, Moore D, van Dam R, Brown C (2015) Baseline blood values for healthy free-ranging green sea turtles (*Chelonia mydas*) in Puerto Rico. Comp Clin Pathol 24: 567–573. 10.1007/s00580-014-1947-1.

[ref53] Persky ME , Williams JJ, Burks RE, Bowman MR, Ramer JC, Proudfoot JS (2012) Hematologic, plasma biochemistry, and select nutrient values in captive smooth dogfish (*Mustelus canis*). J Zoo Wildl Med 43: 842–851. 10.1638/2012-0002R1.1. 23272352

[ref54] Pillans RD , Franklin CE (2004) Plasma osmolyte concentrations and rectal gland mass of bull sharks *Carcharhinus leucas*, captured along a salinity gradient. Comp Biochem Physiol A Mol Integr Physiol 138: 363–371. 10.1016/j.cbpb.2004.05.006. 15313492

[ref55] R Core Team (2020). R: a language and environment for statistical computing. Available at: https://www.r-project.org/. [Accessed September 16, 2021]

[ref56] Ritter MC , Dempsey ME (1971) Specificity and role in cholesterol biosynthesis of a squalene and sterol carrier protein. J Biol Chem 246: 1536–1539. 10.1016/S0021-9258(19)77003-3. 5545095

[ref57] Romero LM , Beattie UK (2022) Common myths of glucocorticoid function in ecology and conservation. J Exp Zool 337A: 7–14. 10.1002/jez.2459 33819389

[ref58] Rosales XQ , Chu M-L, Shilling C, Wall C, Pastores GM, Mendell JR (2008) Fidelity of gamma-glutamyl transferase (GGT) in differentiating skeletal muscle from liver damage. J Child Neurol 23: 748–751. 10.1177/0883073808314365. 18354148

[ref59] Serena F (2005) Field Identification Guide to the Sharks and Rays of the Mediterranean and Black Sea. Food and Agriculture Organization of the United Nations, Rome, Italy

[ref60] Skomal GB , Mandelman JW (2012) The physiological response to anthropogenic stressors in marine elasmobranch fishes: a review with a focus on the secondary response. Comp Biochem Physiol A Mol Integr Physiol 162: 146–155. 10.1016/j.cbpa.2011.10.002. 22008842

[ref61] Spargo AL (2001) The physiological effects of catch and release angling on the post-release survivorship of juvenile sandbar sharks (*Carcharhinus plumbeus*) (Master's thesis). University of Rhode Island, Kingston, RI.

[ref62] Speers-Roesch B , Treberg JR (2010) The unusual energy metabolism of elasmobranch fishes. Comp Biochem Physiol A Mol Integr Physiol 155: 417–434. 10.1016/j.cbpa.2009.09.031. 19822221

[ref63] Stevens JD , Bonfil R, Dulvy NK, Walker PA (2000) The effects of fishing on sharks, rays, and chimaeras (chondrichthyans), and the implications for marine ecosystems. ICES J Mar Sci 57: 476–494. 10.1006/jmsc.2000.0724.

[ref64] Walsh CJ , Luer CA (2004) Elasmobranch hematology: identification of cell types and practical applications. In M Smith, D Warmolts, D Thoney, R Hueter, eds, The Elasmobranch Husbandry Manual: Captive Care of Sharks, Rays, and Their Relatives, Ohio Biological Survey Special Publication, Columbus, OH, pp. 307–323

[ref65] Wells RMG , McIntyre RH, Morgan AK, Davie PS (1986) Physiological stress responses in big gamefish after capture: observations on plasma chemistry and blood factors. Comp Biochem Physiol A Mol Integr Physiol 84: 565–571. 10.1016/0300-9629(86)90366-X. 2874936

[ref66] Whiting SD , Guinea ML, Limpus CJ, Fomiatti K (2007) Blood chemistry reference values for two ecologically distinct populations of foraging green turtles, eastern Indian Ocean. Comp Clin Pathol 16: 109–118. 10.1007/s00580-006-0646-y.

[ref67] Wosnick N , Bornatowski H, Ferraz C, Afonso A, Sousa Rangel B, Hazin FHV, Freire CA (2017) Talking to the dead: using post-mortem data in the assessment of stress in tiger sharks (*Galeocerdo cuvier*) (Péron and Lesueur, 1822). Fish Physiol Biochem 43: 165–178. 10.1007/s10695-016-0276-5. 27549099

[ref68] Wosnick N , Chaves AP, Niella YV, Takatsuka V, Hazin FHV, Nunes JLS, Morick D (2020) Physiological impairment as a result of bile accumulation in an apex predator, the tiger shark (*Galeocerdo cuvier* Péron & Lesueur, 1822). Animals 10: 2030. 10.3390/ani10112030. 33158068 PMC7694183

[ref69] Yang S-T , Kreutzberger AJB, Lee J, Kiessling V, Tamm LK (2016) The role of cholesterol in membrane fusion. Chem Phys Lipids 199: 136–143. 10.1016/j.chemphyslip.2016.05.003. 27179407 PMC4972649

[ref70] Yang T , Haas HL, Patel S, Smolowitz R, James MC, Williard AS (2019) Blood biochemistry and haematology of migrating loggerhead turtles (*Caretta caretta*) in the Northwest Atlantic: reference intervals and intra-population comparisons. Conserv Physiol 7: coy079. 10.1093/conphys/coy079. 30746149 PMC6366141

[ref71] Zemah Shamir Z , Zemah Shamir S, Tchernov D, Scheinin A, Becker N (2019) Shark aggregation and tourism: opportunities and challenges of an emerging phenomenon. Int J Sustain Dev World Ecol 26: 406–414. 10.1080/13504509.2019.1573769.

