# Supplementary material for: First report on the serum chemistry and haematology of free-ranging dusky (Carcharhinus obscurus) and sandbar (Carcharhinus plumbeus) sharks in the eastern Mediterranean Sea
Source: Conserv Physiol. 2023 May 30;11(1):coad037. doi: 10.1093/conphys/coad037 (PMC10230282; doi:10.1093/conphys/coad037)
Supplement: Web_Material_coad037 [file web_material_coad037.pdf]

Supplementary material

Table S1: Shark, effort, environmental, and serum chemistry data of wild dusky (*Carcharhinus obscurus*) and sandbar (*C. plumbeus*) sharks from the eastern Mediterranean Sea in conventional units

Shark No.	Capture Date	Catch Time	Salinity (ppt)	Temp. (°C)	Species	Sex	Fork Length (m)	Hook Time (min)	Urea (mg/dL)	Total Protein (g/dL)	AST (U/L)	GGT (U/L)	Bilirubin (mg/dL)	Glucose (mg/dL)	Total Chol. (mg/dL)	Trig. (mg/dL)	Amy. (U/L)	CK (U/L)	P (mg/dL)	Cl (mmol/L)	Ca (mg/dL)	Na (mmol/L)	K (mmol/L)
0043	23-03-16	11:51			C. obscurus	F	2.65	36	2816	4.3	54			78	38	14		4589	4.7	252	15.3	330	6.2
0001	17-01-17	11:50	40.0	22.2	C. obscurus	F	2.26	50						54	78		50						
0003	24-01-17	12:28	38.3		C. obscurus	F	2.35	37		4.46	118	137	0.58	79	144	106	99	702	9.3	267	14.77	264	5.6
0005	21-02-17	10:45	40.0	21.3	C. obscurus	F	2.5	39		4.69	61	48	0.36	68	146	137	77	4486	7.4	301	15.41	280	
0007	23-02-17	11:43	40.0	21.9	C. plumbeus	M	1.44	28						54	112	72	32						
0008	28-02-17	09:29	39.3	20.7	C. obscurus	F	2.42	43			138	28	0.95	78	130	115	114	4852	8.2	296	14.33	271	5.3
0010	08-03-17	09:46	39.0	21.5	C. plumbeus	M	1.44	24	3043.4	3.84	99	131	0.02	63	119	68	107	9851	6	299	14.64	279	6
0011	08-03-17	12:18	38.9	22.5	C. obscurus	F	3.43	27	2971.1	4.81		131	0.12	75	120	75	100	646	5	294	15.03	282	4.1
0001	23-03-17	09:37	39.6	24.2	C. obscurus	F	2.31	49			20					148		11503					
0012	28-03-17	11:41	41.0	23.8	C. obscurus	F	2.59	32	2266.6	3.32	27	224	0.38	71	156	146	133	1730	5.8	299	14.95	287	5.1
0013	06-04-17	09:42	40.5	24.8	C. plumbeus	M	1.6	34	2645	3.87	85	77	0.1	90	135	84	8	10340	5.3	329	16.19	293	4.3
0014	06-04-17	12:58	40.3	26.0	C. plumbeus	M	1.48	27	2738	3.84	179	268	0.13	67	149	106	143	83546	8	297	14.4	277	6.1
0016	12-12-17	14:34			C. obscurus	F	2.2	35	2350.6	3.65	20			95	50	14	2	1705	5.1	319	16.22	303	4.6
0018	27-12-17	07:17			C. obscurus	F	2.59	43		4.21	59	27	0.02	78	93	47	25	9332	5.6	312	15.79	297	4.9
0019	27-12-17	10:43			C. obscurus	F	2.52	38		4.72	44	72	0.06	76	104	65	188	5624	5.4	297	15.95	289	4
0021	01-02-18	13:00	39.8	21.9	C. obscurus	F	2.59	40	2746.5	3.38	19		0.01	110	28	19		537	5.1	310	17.15	301	4.4
0022	06-02-18	13:36	39.6	21.7	C. obscurus	F	2.27	34	2416.4	4.11	14		0.2	90	63	26	3	1766	5.5	306	15.61	290	4.4

0023	12-03-18	13:00	39.7	24.4	C. plumbeus	M	1.39	25	2456.4	4.08	25		0.1	63	86	22		885	5.8	295	16.17	281	5.1
0025	14-03-18	10:55	39.6	24.4	C. plumbeus	M	1.51	30	2372.9	3.88	82		0.01	68	65	35		20154	6.8	285	15.43	280	3.9
0026	28-03-18	08:52	32.7	24.2	C. obscurus	F	2.46	52	2462.6	3.67	109			106	40	24		17836	7.8	301	14.73	290	5.2
0027	28-03-18	11:59	36.1	23.5	C. plumbeus	M	1.47	36	2440.7	3.89	41		0.01	68	102	41		12871	6.2	294	15.73	287	3.9
0028	02-04-18	11:33	40.0	22.8	C. obscurus	F	2.47	53	2437.9	4.05				78	62	72	169						
0031	27-11-18	16:02	40.8	26.9	C. obscurus	F	2.38	57	2140.1	4.3		31			116.6	77.8	14	1078		319.5	16.56	305	3.15
0032	16-12-18	13:03	39.1	22.2	C. obscurus	F	2.42	44	2092.4	4.16	74	2.9			73.5	26		7371		310.1	16.5	302	4.6
0033	21-01-19	12:30	39.3	22.1	C. plumbeus	M	1.49	35	2130.7	4.22	51	2.9		46	134	28	1	4147	7.98	316.9	15.8	301	4.02
0034	20-02-19	10:27	37.7	18.0	C. obscurus	F	2.46	47	2269.8	3.79		77	0.069	108.6	79	47.5	8	2508	4.91	310.1		299	3.85
0035	20-02-19	11:41	37.7	18.0	C. plumbeus	M	1.6	39				17	0.044	53.5	101.1	46.7							
0036	24-02-19	18:54	39.5	22.0	C. plumbeus	M	1.54	34	2200.8	3.95		105		94	134.4	64	7	5856	6.15	291	16.1	282	4.99
0037	25-02-19	15:45	39.7	21.7	C. obscurus	F	2.45	62	2111.8	4.11		2.9		111	64.5	20							
0038	11-03-19	12:50	39.9	24.0	C. plumbeus	M	1.6	35	2054.1	4.47	67	2.9		63	108.4	41		3681	5.85	309.4	16.8	296	3.45
0040	20-03-19	17:45			C. obscurus	M	2.24	74		5.49	51	7		86	96.8	39	375	7348	7.22		14.1		4.77
0050	24-04-19	14:45	37.0	19.2	C. plumbeus	M	1.53	39	2357.2	3.555	29		0.0805	67.5	153.55	26.75	0.5	7396.5	5.265	288.15		280	
0051	25-11-19	16:25	40.0	25.6	C. obscurus	F	2.15	54		4.45	44	2.9		91	42.4	9	1	5605	5.7	319.6	16.9	307	4.53
0052	14-01-20	13:11	37.2	23.3	C. obscurus	F	2.54	67	2033.9	4.19				84	67.4	64		9565					
0053	15-01-20	06:32	37.3	22.9	C. obscurus	F	2.33	52	2455.2	3.39	26	2.9		91	36.8	7	1	2750	4.92	301.3	15.4	287	3.63
0053	05-02-20	07:50	38.2	23.6	C. obscurus	F	2.33	41															
0054	17-02-20	11:41	36.2	23.1	C. plumbeus	M	1.46	33	2407.5	4.42	25	2.9		66	72.3	32	3	7390	4.11	281.9	14.8	273	2.72
0055	17-02-20	13:22	39.0	23.3	C. plumbeus	M	1.54	40	2340.2	3.78	21	2.9		60	93.7	44	2	3025	5.82	293.7	16	288	3.31
0056	04-03-20	09:30	35.6	24.1	C. obscurus	F	2.4	65	2146.7	3.43	199	2.9		91	65.3	17		34048	7.54	301.8	15.4	293	4.63

Abbreviations: Temp., Temperature; Total Chol., Total Cholesterol; Trig., Triglycerides; and Amy., Amylase

Table S2: Comparison of the current results for wild dusky (*Carcharhinus obscurus*) and sandbar (*C. plumbeus*) sharks from the eastern Mediterranean Sea with published serum chemistry analytes in conspecifics and other shark species elsewhere

Species	Statists	Urea		Bilirubin		Glucose		Triglycerides		Cholesterol		Total Protein		Amylase		GGT		CK		AST		K		P		Cl		Na		Ca	
		Co	Cp	Co	Cp	Co	Cp	Co	Cp	Co	Cp	Co	Cp	Co	Cp	Co	Cp	Co	Cp	Co	Cp	Co	Cp	Co	Cp	Co	Cp	Co	Cp	Co	Cp
***C. plumbeus*[1]**	test					t																									
	P					0.405																									
***C. plumbeus*[2]**	test	t	t																			t	t				t	t	t		
	P	0.601	0.879																			<0.001	0.015				<0.001	<0.001	<0.001		
***C. plumbeus*[3]**	test					t																t	t				t	t	t		
	P					0.02																0.109	0.121				<0.001	0.005	0.28		
***C. obscurus*[3]**	test					**t**																t	t				t	t	t		
	P					**<0.001**																<0.001	0.002				<0.001	<0.001	0.004		
***C. limbatus*[1]**	test					t																									
	P					0.022																									
***C. limbatus*[4]**	test					W	W															W	W	W	W	W	W	W	W	W	W
	P					<0.001	0.346															0.004	0.505	0.001	0.004	<0.001	<0.001	<0.001	0.003	<0.001	<0.001
***C. limbatus*[5]**	test								t	t	t																				
	P								<0.001	0.002	<0.001																				
***C. leucas*[4]**	test					W	W															W	W	W	W	W	W	W	W	W	W
	P					<0.001	0.006															<0.001	0.004	0.001	<0.001	<0.001	<0.001	0.133	0.196	<0.001	<0.001
***C. leucas*[5]**	test								t	t	t																				
	P								0.005	0.008	<0.001																				
***Galeocerdo cuvier*[5]**	test								t																						
	P								0.003																						
***Rhizoprionodon terraenovae*[6]**	test					W	W			W	W							W	W	W	W	W	W	W	W					W	W
	P					<0.001	0.001			0.553	0.008							<0.001	<0.001	0.006	0.023	<0.001	0.009	0.025	0.005					<0.001	<0.001
***Sphyrna mokarran*[1]**	test					t																									
	P					<0.001																									
***Ginglyostoma cirratum*[1]**	test					t																									
	P					<0.001																									
***Ginglyostoma cirratum*[5]**	test								t	t	t																				
	P								<0.001	<0.001	<0.001																				
***Ginglyostoma cirratum*[7]**	test											t	t																		
	P											<0.001	0.002																		
***Sphyrna tiburo*[4]**	test					W	W															W	W	W	W	W	W	W	W	W	W
	P					<0.001	0.001															<0.001	0.004	0.008	0.004	<0.001	<0.001	<0.001	0.003	0.002	0.002
***Sphyrna tiburo*[6]**	test					W	W	W	W	W	W							W	W	W	W	W	W	W	W					W	W
	P					<0.001	0.001	0.018	0.003	0.006	0.67							<0.001	<0.001	0.142	0.168	<0.001	0.008	0.016	0.005					<0.001	<0.001
***Sphyrna tiburo*[8]**	test	W	W			W	W					W	W					W	W	W	W	W	W	W	W	W	W	W	W	W	W
	P	0.01	0.003			<0.001	0.001					<0.001	0.003					<0.001	<0.001	0.276	0.23	<0.001	0.004	<0.001	<0.001	0.021	0.071	0.002	0.423	<0.001	0.006
***Carcharias taurus*[9]**	test	t	t	W	t	t	W	W	t	t	t	t	t					W	W	W	W	t	t	W	t	W	t	t	t	t	t
	P	0.134	0.058	0.275	0.158	<0.001	0.001	0.017	0.003	0.002	<0.001	<0.001	0.002					<0.001	<0.001	0.012	0.051	0.036	0.073	0.636	0.138	<0.001	<0.001	<0.001	<0.001	0.85	0.857
***Rhincodon typus*[10]**	test				t	t						t	t									t	t		t		t	t	t	t	t
	P				<0.001	<0.001						<0.001	0.001									<0.001	0.005		<0.001		<0.001	0.018	0.762	<0.001	<0.001
***Squalus acanthias*[6]**	test					W	W	W	W	W	W									W	W	W	W	W	W					W	W
	P					<0.001	0.001	0.032	0.008	0.057	0.091									<0.001	0.004	0.004	0.505	<0.001	<0.001					<0.001	<0.001
***Squalus acanthias*[11]**	test	W	W			t																t	t				t	t	t		
	P	<0.001	<0.001			<0.001																0.008	0.455				<0.001	<0.001	<0.001		

Abbreviations: D, dusky shark; S, sandbar shark; t, one sample t-test; and W, one sample Wilcoxon test

Black text denotes no significant difference between our data and the published value; Red text denotes our results are significantly higher than the published value; Green text denotes our results are significantly lower than the published value

[1]Jerome et al. 2017; [2]Brill et al. 2008; [3]Marshall et al. 2012; [4]Manire et al. 2001; [5]Gallagher et al. 2017; [6]Haman et al. 2012; [7]AtallahBenson et al. 2020; [8]Harms et al. 2002; [9]Otway 2015; [10]Dove et al. 2010; [11]Mandelman and Farrington 2007

Table S3: Hematological data of wild dusky (*Carcharhinus obscurus*) and sandbar (*C. plumbeus*) sharks from the eastern Mediterranean Sea

Shark No.	Species	Stain	Lymph. (%)	Mono. (%)	Eosin. (%)	Hetero. (%)	Neutro. (%)	Y. Gran. (%)	Gran. Thromb. (%)	Thromb. (%)	PCV (%)
0031	*C. obscurus*	Modified Wright's	36	4	1	9	5	0	4	41	
0032	*C. obscurus*	Diff-Quik	15	1	9	22	2	0	4	47	37
0034	*C. obscurus*	Modified Wright's	20	3	2	6	5	0	20	44	40
0040	*C. obscurus*	Modified Wright's	17	4	3	22	3	0	15	36	46
0051	*C. obscurus*	Modified Wright's	8	2	5	5	2	1	14	63	35
0053	*C. obscurus*	Diff-Quik	5	2	2	26	12	0	0	53	37
0056	*C. obscurus*	Modified Wright's	23	1	5	4	2	0	27	38	37
0033	*C. plumbeus*	Diff-Quik	20	2	6	5	7	0	0	60	45
0035	*C. plumbeus*	Modified Wright's	25	2	6	2	1	0	0	64	48
0036	*C. plumbeus*	Modified Wright's	29	3	7	5	2	0	14	40	34

0038	*C. plumbeus*	Modified Wright's	20	4	8	3	5	0	13	47	49
0050	*C. plumbeus*	Diff-Quik	19	2	11	9	5	0	10	44	33
0054	*C. plumbeus*	Modified Wright's	27	1	9	8	1	1	17	36	47
0055	*C. plumbeus*	Modified Wright's	37	2	8	4	2	0	13	34	57

Abbreviations: Lymph., Lymphocytes; Mono., Monocytes; Eosin., Eosinophils; Hetero., Heterophils; Neutro., Neutrophils; Y. Gran., Young Granulocytes; Gran. Thromb., Granulated Thrombocytes; Thromb., Thrombocytes; and PCV, Packed Cell Volume
